# Association of *Helicobacter pylori* Infection with Glycemic Control in Patients with Diabetes: A Meta-Analysis

**DOI:** 10.1155/2014/250620

**Published:** 2014-05-08

**Authors:** Chika Horikawa, Satoru Kodama, Kazuya Fujihara, Yoko Yachi, Shiro Tanaka, Akiko Suzuki, Osamu Hanyu, Hitoshi Shimano, Hirohito Sone

**Affiliations:** ^1^Department of Health and Nutrition, Faculty of Human Life Studies, University of Niigata Prefecture, Niigata, Japan; ^2^Department of Internal Medicine, Faculty of Medicine, Niigata University, 1-754 Asahimachi, Niigata 951-8510, Japan; ^3^Department of Internal Medicine, Institute of Clinical Medicine, University of Tsukuba, Ibaraki, Japan; ^4^Department of Health Management Center, Mito Kyodo General Hospital, Ibaraki, Japan; ^5^Department of Clinical Trial, Design & Management, Translational Research Center, Kyoto University Hospital, Kyoto, Japan

## Abstract

*Objective*. To assess the association between *Helicobacter pylori* (HP) infection and glycemic control in patients with diabetes through a meta-analytic approach. *Research Design and Methods*. Electronic literature searches were conducted for cross-sectional studies that examined the hemoglobin A1c (A1C) level by whether patients with diabetes were or were not carriers of HP. Mean differences in A1C between groups with and without HP infection were pooled with a random-effects model. *Results*. Thirteen eligible studies were included in this meta-analysis. Overall, the HP carriers did not have significantly higher A1C levels compared with HP noncarriers (mean difference (95% CI), 0.19% (−0.18 to 0.46), *P* = 0.16). When the analysis was limited to studies targeting patients with type 1 diabetes, there was also no significant difference in A1C (0.69% (−0.31 to 1.68), *P* = 0.18). *Conclusions*. There was insufficient evidence that HP infection worsened glycemic control in patients with diabetes.

## 1. Introduction

Glycemic control is essential in the management of diabetes to prevent diabetic complications as well as their progression, if present [1]. Among various factors that influence the management of the blood glucose level, chronic infections such as periodontal disease [2] or tuberculosis [3] are major causes of worsening of glycemic control or of difficulty in glycemic control.

*Helicobacter pylori* (HP) is a major human bacterial pathogen, the chronic infection of which causes a number of upper gastrointestinal conditions such as chronic gastritis, peptic ulcer disease, gastric malignancy, and gastric mucosa associated lymphoid tissue lymphoma [4]. Moreover, a recent meta-analysis showed that HP infection is 1.3-fold more prevalent in persons with diabetes than in those without diabetes [5]. However, results are inconsistent among studies of the association between chronic HP infection and poor glycemic control in patients with diabetes. The aim of this meta-analysis is to compare glycemic control in patients with diabetes according to the presence or absence of HP.

## 2. Materials and Methods

An electronic literature search was conducted using the search engine Proquest Dialog, which made it possible to search several databases simultaneously. We chose the following databases related to medicine: Biosis (1926 to March 26, 2014), MEDLINE (1950 to March 26, 2014), Embase (1947 to March 26, 2014), PASCAL (1973 to March 26, 2014), and SciSearch (1974 to March 26, 2014). The search equation was produced by combining keywords related to HP and diabetes using the Boolean operator “AND” (Table 1).

Studies were included if they targeted patients with diabetes and provided data on the mean hemoglobin A1c (A1C) level and its corresponding standard error according to whether the patients carried HP. Two of our investigators (Chika Horikawa and Satoru Kodama) independently abstracted these data. Discrepancies were resolved by a third investigator (Hirohito Sone).

Mean differences in A1C between groups with and without HP infection were pooled with a random-effects model using the DerSimonian and Laird method [6]. The extent of between-study heterogeneity was assessed by I-squared statistics [7]. Analyses were repeated for subgroups within which the same study characteristics were shared. Publication bias was statistically assessed by two formal methods: Begg's rank correlation and Egger's regression tests [8, 9]. Two-sided *P* < 0.05 was considered statistically significant with the exception of the test for publication bias where *P* < 0.10 was used [10]. All analyses were conducted with Stata statistical software (version 11, StataCorp, College Station, TX, USA).

## 3. Results

### 3.1. Literature Search and Study Characteristics

**Figure 1:**
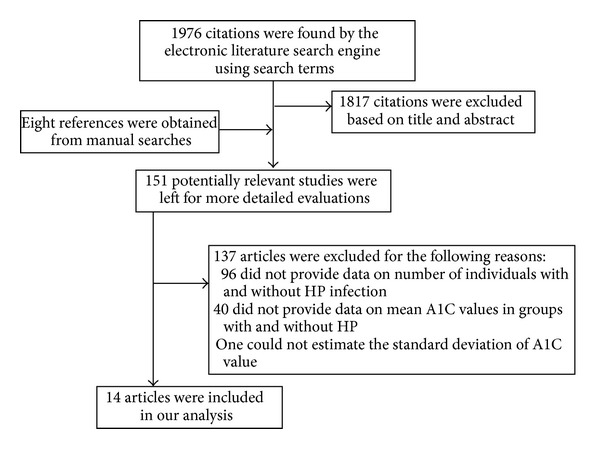
Flow chart of meta-analysis. HP:* Helicobacter pylori*; A1C: hemoglobin A1C.

Figure 1 shows details of the literature search. Of the 1976 citations retrieved from the systematic literature searches, 14 eligible studies [11–24] were obtained.

Characteristics of the 14 selected studies [11–24] comprising 1781 diabetic participants (range, 63–333 participants) and 990 HP-infected participants (range, 11–187 participants) are shown in Table 2. Proportion of men and mean age of study participants ranged from 30.8% to 58.9% and from 11.3 years to 66.3 years, respectively. Seven studies [11–16, 24] included only type 2 diabetes mellitus patients, 5 [19–23] included only type 1 diabetes mellitus patients, and 2 [17, 18] included both type 1 and type 2 diabetes mellitus patients. Four studies [17, 19–21] were conducted in Western countries and 10 studies [11–16, 18, 22–24] took place in non-Western countries. Five of the 14 studies [11–15] used a biopsy for identifying HP infection and the remaining 9 studies [16–24] used other methods such as measurement of HP-specific immunoglobulin G using an enzyme immunoassay and the (13C) urea breath test. Mean duration of diabetes ranged from 2.9 to 16.1 years.

### 3.2. Overall Estimate of Differences in A1C between Diabetic Patients with and without HP Infection

**Figure 2:**
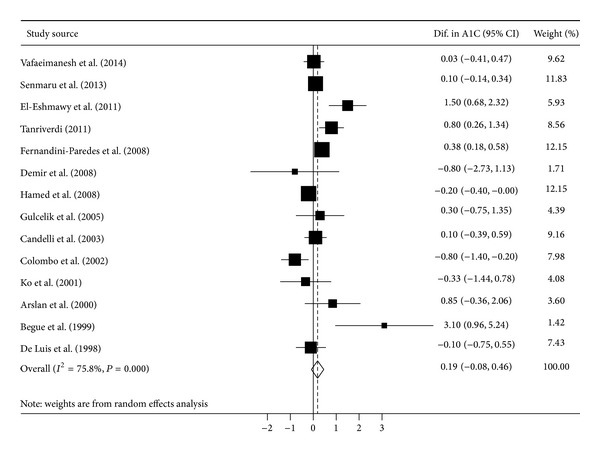
Forest plot of mean differences with corresponding 95% confidence intervals (CIs) in hemoglobin A1C (A1C) for patients with diabetes with* Helicobacter pylori* infection versus those with* Helicobacter pylori* noninfection. Size of squares reflects the statistical weight of each study. Pooled mean difference in A1C is indicated by an unshaded diamond.

A total of 14 datasets were included in this meta-analysis.  Figure 2 shows a forest plot of mean differences in A1C with their corresponding 95% confidence intervals (CIs) for patients with diabetes with HP infection versus those without HP infection. Overall, compared with HP carriers, the HP carriers did not have significantly higher A1C levels (mean difference (95% CI), 0.19% (−0.08 to 0.46), *P* = 0.16). Publication bias was not statistically detected by Egger's test (*P* = 0.45) and Begg's test (*P* = 0.62).

### 3.3. Stratified Analysis

Stratified and metaregression analyses across a number of key study characteristics to explore the origin of the heterogeneity and the influence of the characteristics on study results are shown in Table 3.

When limiting the analysis to the 5 studies that exclusively targeted type 1 diabetes, also no significant difference in A1C was observed (0.69% (−0.31 to 1.68), *P* = 0.18). Including the type of diabetes, other items such as duration of diabetes, geographic region, and methodological features for determination of HP infection did not significantly influence study results.

## 4. Discussion

The current meta-analysis produced insufficient evidence that chronic infection with HP was associated with poor glycemic control in patients with diabetes. This finding seemed contradictory to the biological finding that HP infection stimulates inflammatory responses leading to insulin resistance and persistent hyperglycemia [25] by producing proinflammatory cytokines such as C-reactive protein and interleukin-6 [18, 26]. The speculation for this contradiction is that stimulus by the HP infection of an inflammatory response might be insufficient to worsen glycemic control.

Other speculations may be that (1) chronic hyperglycemia caused by HP infection could have been compensated by increasing doses of antihyperglycemic drugs [21] and (2) the potentially worsening glycemic control might be counterbalanced by “successful” weight control as a result of chronic gastritis and lack of appetite. However, more information on details of treatments, including antihyperglycemic medications, or nutrition surveys of patients with and without HP infection, is necessary to elucidate these speculations.

Major limitation of this meta-analysis is that it did not consider various characteristics other than HP infection that would have influenced glycemic control, such as status of treatment, age, gender, obesity indicators, or smoking status. The difference in A1C levels between patients with and without HP infection might have been attributed more strongly to characteristics for which no included studies matched rather than to HP infection itself. Therefore, this study might have failed to investigate the direct association between HP infection and glycemic control. An additional limitation was that potential publication bias could not be ruled out because of the strong evidence that infection could elevate the blood glucose level even if it was not statistically detected.

To more directly examine the association between HP infection and glycemic control would be to investigate the effect of HP eradication on glycemic control. Unfortunately, we could not conduct a meta-analysis of studies that investigated A1C levels before and after HP eradication because of the insufficient number of such eligible studies [27–31]. Although the results were inconsistent among studies, most studies [27–30] did not indicate the effectiveness of HP eradication on glycemic control with one exception [31]. Nevertheless, further studies would need to investigate the effect of eradication on glycemic control to clarify whether HP infection influences glycemic control.

## 5. Conclusions

This meta-analysis produced insufficient evidence that chronic infection with HP worsened glycemic control in patients with diabetes. More studies are needed to investigate the effect of HP eradication on glycemic control to prove the influence of HP infection on glycemic control.

## Figures and Tables

**Table 1:** Study keywords in this meta-analysis.

S1 [Related to diabetes mellitus]	
Thesaurus terms	
EMBASE (“insulin dependent diabetes mellitus” [NoExp] OR “juvenile diabetes mellitus” [NoExp] OR “diabetic patient” [NoExp] OR	
“diabetes mellitus” [NoExp] OR “maturity onset diabetes mellitus” [NoExp] OR “non insulin dependent diabetes mellitus” [NoExp])	
MEDLINE (“Diabetes Mellitus” [NoExp] OR “Diabetes Mellitus, Type 2” [NoExp] OR	
“Diabetes Mellitus, Type 1” [NoExp])	
text words	
(“diabetes” OR “NIDDM” OR “IDDM” OR “diabetic*”)	
S2 [Related to Helicobacter Pylori]	
Thesaurus terms	
EMBASE (“Helicobacter pylori” [Exp] OR “Helicobacter infection”)	
MEDLINE (“Helicobacter pylori”) [Exp]	
Test word	
“pylori”	
S3 1 AND 2	

[Exp] indicates automatic inclusion of all of the narrower terms under the specified descriptor in the thesaurus hierarchy.

[NoExp] exclusively searches for the specified descriptor.

asterisk (∗) indicates an inflection of the corresponding word.

**Table 2:** Characteristics of studies included in the meta-analysis.

Author	Year	Country	Type of diabetes	Men (%)	Mean age (year)	Duration of diabetes (year)	Mean BMI	Number of participants		Mean HbA1c value (%)		Method for identifying HP infection
								HP infected	HP non-infected	HP infected	HP non-infected	
Vafaeimanesh et al. [24]	2014	Iran	T2DM	36.0	52.5	7.4	29.0	139	82	8.11	8.08	HP-specific IgG using EIA

Senmaru et al. [16]	2013	Japan	T2DM	58.9	66.3	15.1	22.8	187	146	7.4	7.3	HP-specific IgG using EIA

El-Eshmawy et al. [22]	2011	Egypt	T1DM	44.5	19.4	7.3	NA	128	34	8.3	6.8	HP-specific IgA and IgG using EIA

Tanriverdi [15]	2011	Turkey	T2DM	52.7	55.4	2.9	28.2	53	40	6.9	6.1	Biopsy

Fernandini-Paredes et al. [11]	2008	Chile	T2DM	46.7	52.8	8.2	NA	49	26	7.7	7.3	[13C]urea breath test and biopsy

Demir et al. [12]	2008	Turkey	T2DM	32.2	52.0	6.1	NA	87	54	7.9	8.7	Biopsy

Hamed et al. [18]	2008	Egypt	T1DM and T2DM	48.8	47.5	9.2	28.8	68	12	8.1	8.3	HP-specific IgG using EIA

Gulcelik et al. [13]	2005	Turkey	T2DM	30.8	51.9	6.9	26.0	59	19	8.2	7.9	Biopsy

Candelli et al. [19]	2003	Italy	T1DM	54.5	14.8	6.6	20.9	34	87	8.3	8.2	[13C]urea breath test

Colombo et al. [20]	2002	Italy	T1DM	52.9	12.0	5.5	NA	41	97	7.8	8.6	HP-specific IgA and IgG using EIA

Ko et al. [14]	2001	China	T2DM	46.0	49.9	6.2	NA	32	31	8.1	8.4	Biopsy

Arslan et al. [23]	2000	Turkey	T1DM	40.9	12.6	10.7	NA	49	39	11.1	10.2	HP-specific IgG using EIA

Begue et al. [21]	1999	USA	T1DM	50.7	11.3	3.6	20.0	11	60	14.9	11.8	HP-specific IgG using EIA

de Luis et al. [17]	1998	Spain	T1DM and T2DM	50.4	60.2	16.1	28.8	53	74	7.1	7.2	HP-specific IgG using EIA

Abbreviations: HP: *Helicobacter pylori*; EIA: enzyme immunoassay; IgA: immunoglobulin A; IgG: immunoglobulin G; T1DM: type 1 diabetes mellitus; T2DM: type 2 diabetes mellitus.

**Table 3:** Stratified analyses of differences between those with *Helicobacter pylori* (HP) infection versus those without HP infection in hemoglobin A1C level with 95% confidence interval according to key study characteristics.

Variable	Number of data	Mean difference (95% CI), %	*Q* statistics	*I* ^2^ (%)	*P*-value for heterogeneity	Meta-regression
Total	14	0.19 (−0.18 to 0.46)	53.6	75.8%	<0.001	—
Geographic region						
Western	4	0.08 (−0.72 to 0.88)	14.5	79.3%	0.002	Referent
Non-western	10	0.28 (−0.01 to 0.57)	36.6	75.3%	<0.001	0.47
Type of diabetes						
Type 1 diabetes mellitus only	5	0.69 (−0.31 to 1.68)	28.8	86.1%	<0.001	Referent
Type 2 diabetes mellitus was included	9	0.12 (−0.13 to 0.37)	24.8	67.8%	0.002	0.38
Duration of diabetes						
≥8 years	6	0.11 (−0.19 to 0.41)	18.2	78.0%	<0.001	Referent
<8 years	8	0.30 (−0.24 to 0.84)	34.9	77.1%	<0.001	0.73
Method for determination of HP infection						
Biopsy	5	0.40 (0.22 to 0.58)	5.3	24.8%	0.26	Referent
Other methods	9	0.14 (−0.20 to 0.48)	34.4	76.7%	<0.001	0.83
